# Management of Low Colorectal/Coloanal Anastomotic Leak: Results of a French National Intergroups Practice Survey (FRENCH‐GRECCAR‐SFCD)

**DOI:** 10.1002/wjs.12634

**Published:** 2025-05-21

**Authors:** Clément Pastier, Wafa Ben Hmida, Jérémie H. Lefèvre, Quentin Denost, Lilian Schwarz, Stéphane Berdah, Eddy Cotte, Mehdi Karoui, Léon Maggiori, Solafah Abdalla, Antoine Brouquet, Stéphane Benoist

**Affiliations:** ^1^ Department of Oncologic and Digestive Surgery Bicêtre Hospital Assistance Publique‐ Hôpitaux de Paris Le Kremlin‐Bicêtre France; ^2^ Department of Colorectal Surgery Saint‐Antoine Hospital Assistance Publique Hôpitaux de Paris Paris France; ^3^ Department of General and Digestive Surgery Bordeaux Colorectal Institute Bordeaux France; ^4^ Department of Digestive Surgery Rouen University Hospital Rouen France; ^5^ Department of Digestive Surgery Nord Hospital Assistance Publique‐Hôpitaux de Marseille Marseille France; ^6^ Department of General and Surgical Oncology Hospices Civils de Lyon Lyon France; ^7^ Department of Gastro‐Intestinal and Oncologic Surgery Georges Pompidou European Hospital Paris France; ^8^ Department of General‐Endocrine and Digestive Surgery Saint Louis Hospital Paris France

**Keywords:** coloanal anastomotic fistula, colorectal anastomotic fistula, stoma

## Abstract

**Aims:**

Anastomotic leakage (AL) impacts short‐term and long‐term outcomes after colorectal surgery, yet no consensus exists regarding its diagnosis and management. The aim was to establish a proactive consensus‐based approach for diagnosing and treating AL following rectal cancer surgery through a national survey.

**Methods:**

A questionnaire was designed to assess 24 clinical scenarios related to the diagnosis and management of fistulas in low colorectal (LCA) or coloanal anastomosis (CAA) with a diverting ileostomy.

**Results:**

A total of 203 surgeons from three surgical societies participated. Consensus was reached on four key indicators warranting further investigation of AL: CRP > 250 mg/L, fever ≥ 38.5°C, tachycardia > 100 bpm, and diffuse abdominal pain. In the presence of any warning sign, 87% recommended an urgent contrast‐enhanced abdominopelvic CT scan without routine rectal contrast as the first‐line diagnostic tool. Isolated extra‐digestive air bubbles or uncollected effusions without air bubbles were managed with antibiotics (61%–78%). A perianastomotic collection required an anal examination under general anesthesia (70%). For treatment, transanal drainage (56%) was preferred over image‐guided percutaneous drainage, combined with endoluminal vacuum therapy and at least 7 days of antibiotics (97%). Drain removal was recommended (64%) when imaging confirmed the absence of residual collection.

**Conclusions:**

This national survey established a consensus‐driven proactive management algorithm for LCA/CAA fistulas. Further validation controlled trial is needed to confirm the effectiveness in reducing AL‐related complications.

## Graphical abstract

Anastomotic leakage (AL) impacts short‐term and long‐term outcomes after colorectal surgery, yet no consensus exists regarding its diagnosis and management. The aim was to establish a proactive consensus‐based approach for diagnosing and treating AL following rectal cancer surgery through a national survey. A questionnaire was designed to assess 24 clinical scenarios related to the diagnosis and management of fistulas in low colorectal (LCA) or coloanal anastomosis (CAA) with a diverting ileostomy.

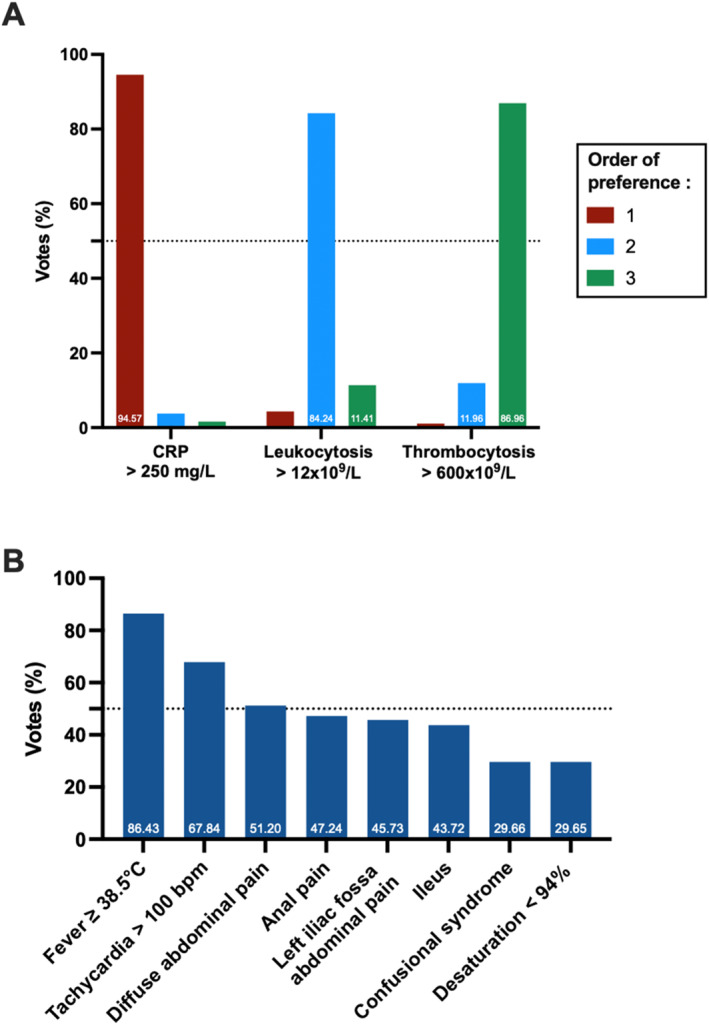

**Summary**

- What does this paper add to the literature?
  ◦ This study represents a large‐scale practice survey conducted within the community of French academic colorectal surgeons. Its primary aim is to capture routine clinical practices regarding diagnostic and therapeutic strategies used in the management of this complication. It establishes a consensus‐based proactive management algorithm for anastomotic leak, which will be evaluated in controlled trial in the near future.

**Abbreviations**
CAA: coloanal anastomosis
CRP: c‐reactive protein
CT: computed tomography
LCA: low colorectal anastomosis

## 1 Introduction

Despite substantial advancements in surgical techniques, anastomotic leakage (AL) remains the most frequent and severe complication following rectal cancer surgery. This complication significantly affects long‐term functional and oncological outcomes as well as patients' quality of life [1, 2, 3, 4, 5].

Although reported incidence rates can vary across studies, AL occurs in approximately 10%–15% of patients undergoing total mesorectal excision (TME) with low colorectal (LCA) or coloanal anastomosis (CAA), even in high‐volume expert centers [6, 7, 8, 9, 10]. The risk factors for AL have been extensively investigated, with most being nonmodifiable parameters such as sex, obesity, tumor location, and anastomotic level. Consequently, few preventive measures are available beyond the routine use of a diverting ileostomy [11, 12, 13, 14, 15, 16].

In patients with a low anastomosis and diverting stoma, AL most commonly presents as a perianastomotic abscess, diagnosed via a CT scan as a localized fluid collection around the anastomosis [7, 17]. Management options range from antibiotics to various types of drainages (image‐guided percutaneous, surgical, or endoscopic), and in some cases, additional surgical procedures aim at promoting anastomosis healing [18, 19, 20, 21, 22]. However, none of these approaches has demonstrated clear superiority over the others, and regardless of the initial chosen strategy, the overall anastomotic preservation rate in cases of AL remains approximately 50% [15, 23, 24, 25, 26, 27].

Recent data suggest that the delay between AL diagnosis and treatment often exceeds 2 weeks [24], a delay that may negatively impact patient's outcomes. Interestingly, early treatment of AL may be associated with improved healing rates and a higher likelihood of anastomotic preservation [19, 27]. However, the often nonspecific clinical presentation of AL can lead to underestimation or misdiagnosis, contributing to delays in therapeutic intervention. In a comparable clinical setting, pancreatic surgeons have demonstrated that implementing a standardized diagnostic and therapeutic algorithm could improve outcomes in patients with postoperative pancreatic fistula [28].

Recognizing the benefits of a structured approach, we conducted a national practice survey to establish a proactive and consensual strategy for the diagnosis and management of AL following rectal cancer surgery.

## 2 Methods

### 2.1 Survey Design (Figure S1)

The study was conducted using a structured questionnaire comprising 24 clinical scenarios related to the diagnosis and management of AL following LCA or CAA with a diverting ileostomy (Figure S1). Each scenario was developed and validated by the scientific committee of three surgical scientific societies FRENCH (Fédération de Recherche EN CHirurgie), GRECCAR (Groupe de Recherche En Chirurgie du CAncer du Rectum), and SFCD (Société Française de Chirurgie Digestive). The questionnaire included 11 questions focusing on diagnostic modalities and 13 questions addressing therapeutic management. For each scenario, respondents were asked to select one or multiple responses among 2–8 choices. In one specific item, respondents were required to rank options according to their preferred management strategy.

Respondents were all members of one of the three surgical societies FRENCH, GRECCAR, or SFCD. The GRECCAR members were colorectal surgeons, whereas members of the other scientific associations included general digestive surgeons.

Each participating association disseminated the survey via e‐mail, providing a link to a dedicated online platform (https://www.surveymonkey.com/). Participation was restricted to board‐certified practicing surgeons. In addition, in the electronic mail, it was asked to the physicians to participate to the survey only if they were routinely involved in treatment of rectal cancer. Upon accessing the questionnaire, respondents were required to declare their affiliated institution and the surgical association that they were representing. They could choose to answer all or only a subset of the questions, and all responses remained anonymous. To prevent duplicate submissions, participants affiliated with multiple associations were allowed to respond only once. The survey remained open for 2 months, from April 2023 to May 2023. During this period, two reminder e‐mails were sent to each participating surgical association members to encourage responses. The survey was conducted in accordance with the CROSS and CHERRIES guidelines for survey‐based research [29, 30].

### 2.2 Statistical Analysis

Responses collected via the online platform were compiled and analyzed using Microsoft Excel. A consensus was considered reached when more than 50% of respondents selected the same answer. Consensus strength was categorized as “moderate” when agreement was more than 50% but less than 60%, “strong” when agreement was between 61% and 75%, and “very strong” if more than 75% of participants recommended the same management approach. Statistical analysis of the response differences was performed using the chi‐squared or Fisher's nonparametric test as appropriate.

## 3 Results

The questionnaire has been sent to 313 surgeons. A total of 203/313 (64.8%) surgeons from three surgical societies participated in the survey. The respondents had a high level of experience, with 112 (56%) having more than 10 years of practice, and 46 (23%) having less than 5 years of experience. For each scenario, at least 198 answers were obtained.

### 3.1 Diagnostic Modalities

**FIGURE 1:**
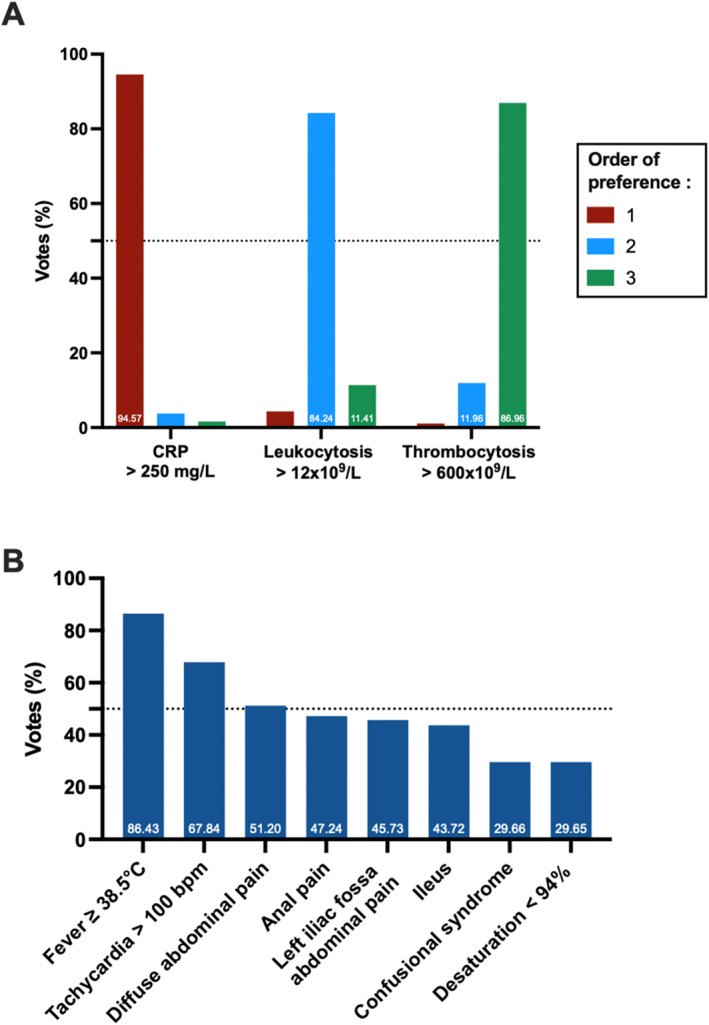
Ranking of symptoms suggesting anastomotic fistula by order of importance. (A) Biomarkers and (B) clinical symptoms.

The survey included 11 questions related to diagnostic approaches (Figure S1). There was a very strong consensus (96%) in favor of a serial measurement of C‐reactive protein (CRP) blood level every other day after rectal surgery, whereas only 20% of surgeons reported using postoperative procalcitonin (PCT) monitoring. Among biochemical markers, only a CRP level exceeding 250 mg/L postoperatively was considered relevant for AL diagnosis. In contrast, fewer than 5% of surgeons considered hyperleukocytosis > 12 × 10^9^/L or thrombocytosis > 600 × 10^9^/L as significant indicators (Figure 1A).

Between postoperative day 1 and day 5, consensus was reached on four individual warning signs that should prompt investigation for AL (Figure 1B): CRP > 250 mg/L (86.9%, very strong consensus), fever ≥ 38.5°C (86.4%, very strong consensus), tachycardia > 100 bpm (67.8%, strong consensus), and diffuse abdominal pain (51.2%, moderate consensus). Additionally, a CRP level exceeding 150 mg/L beyond postoperative day 5 was also considered as indication for further investigation for AL (52%, moderate consensus). In the presence of any of these warning signs, there was a strong consensus (60%–87%) to perform an urgent abdominopelvic CT scan as the first‐line diagnostic modality to confirm AL diagnosis, rather than an anal examination under anesthesia (3%–18%) or a bedside digital rectal examination (3%–13%).

**FIGURE 2:**
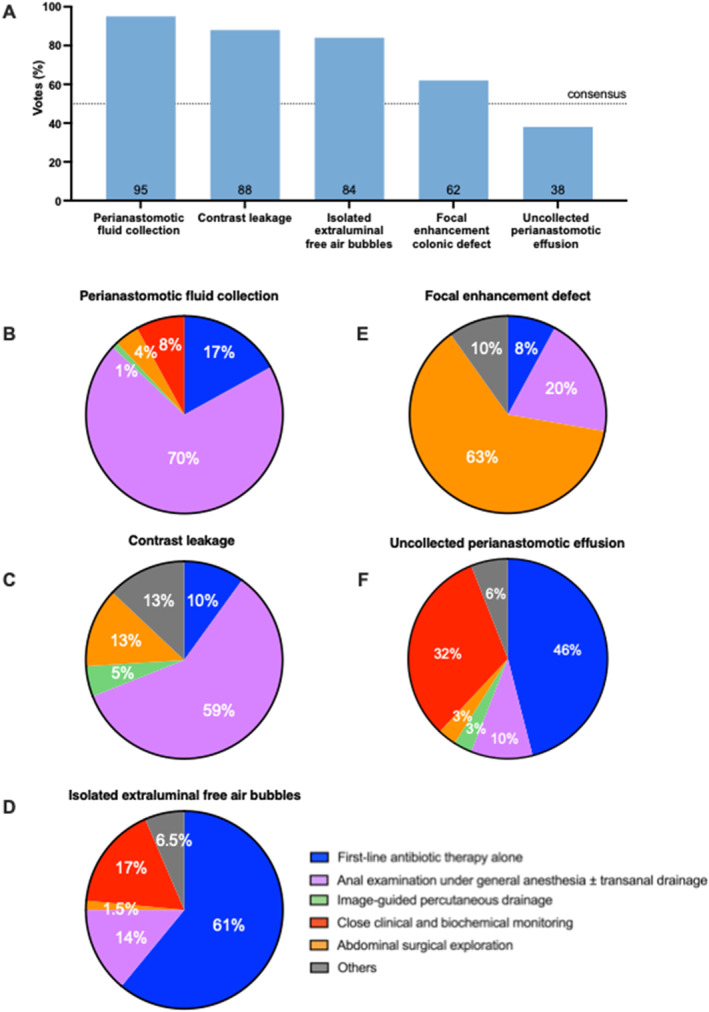
An overview of the therapeutic modalities according to CT scan findings. (A) Radiological signs indicating an anastomotic leak diagnosis. (B) Management of perianastomic fluid collection. (C) Management of contrast leakage. (D) Management of isolated extradigestive air bubbles. (E) Management of focal enhancement defect on the colon wall. (F) Management of uncollected perianastomotic effusion.

Regarding imaging techniques, there was a very strong consensus (95%), in favor of performing a CT scan with intravenous contrast, but without routine rectal contrast (75%). A strong consensus (64%) indicated that a CT scan was reliable for diagnosing AL only from postoperative day 3 onward, with a limited diagnostic value before this time. Specific radiological findings associated with AL that reached very strong consensus included perianastomotic fluid collection (95%), extraluminal air bubbles (84%), and contrast extra‐anastomotic leakage (88%). A strong consensus (62%) was reached regarding the relevance of a focal enhancement defect in the colon wall at or upstream of the anastomosis. Conversely, uncollected perianastomotic effusion was not considered a definitive radiological sign of AL (38%) (Figure 2).

### 3.2 Therapeutic Management (Figure 2)

In cases of isolated extraluminal free air bubbles without other radiological signs of AL, moderate consensus (61%) supported first‐line antibiotic therapy alone, without additional drainage. Similarly, first‐line antibiotic therapy and surveillance (78%) was preferred for uncollected perianastomotic effusion without extraluminal air bubbles. In the presence of a perianastomotic fluid collection, strong consensus (70%) supported performing an anastomosis examination under general anesthesia (AEG) before deciding on potential drainage. This approach was also favored by 59% of respondents when contrast leakage was detected on a CT scan.

A strong consensus (63%) favored surgical abdominal exploration (either laparoscopic or open) in cases of focal enhancement defect in the colon wall at or upstream of the anastomosis. If AL requiring drainage was confirmed, moderate consensus (56%) favored transanal drainage over image‐guided percutaneous drainage (44%) when both options were technically feasible.

In cases of AL confirmed using AEG or endoscopic examination, moderate consensus (51%) supported endoluminal vacuum therapy as the preferred drainage technique (51%). Regardless of the drainage method, very strong consensus (97%) recommended adjunctive antibiotic therapy. Regarding antibiotic therapy duration, strong consensus (69%) favored a treatment course of 7–10 days. For transanal or image‐guided percutaneous drainage, the consensus was to remove the drainage system if follow‐up imaging confirmed resolution of the fluid collection (64%). If endoluminal vacuum therapy was used, strong consensus (67%) supported a maximum of three sponge changes.

During AEG, if the AL affected more than 50% of the anastomosis circumference, 81% of surgeons indicated that they would attempt transanal drainage, despite recognizing a low probability of healing. Furthermore, strong consensus (71%) indicated that no additional therapeutic intervention was necessary after removal of transanal drainage, regardless of the drainage technique used.

### 3.3 Algorithm Development

**FIGURE 3:**
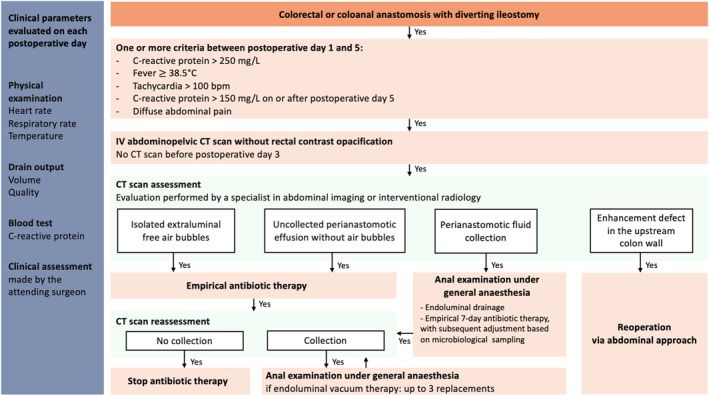
Multimodal algorithm for early detection and management of complications after colorectal or coloanal anastomotic leakage with diverting ileostomy.

Based on these findings, a consensual algorithm was established for the early diagnosis and management of AL following LCA or CAA with diverting ileostomy (Figure 3). Only answers that achieved at least moderate consensus were incorporated into the algorithm. According to this framework, the presence of fever, tachycardia, or diffuse abdominal pain should prompt a CT scan, with CRP as the only relevant biochemical marker. Isolated extraluminal air bubbles or uncollected perianastomotic effusion without air bubbles should be managed with empirical antibiotic therapy alone. In contrast, a perianastomotic fluid collection should warrant an anastomotic anal examination under general anesthesia followed by endoluminal drainage. The presence of a focal enhancement defect on the colon wall on or upstream of the anastomosis should prompt surgical exploration via an abdominal approach. Endoluminal drainage should be monitored with a follow‐up CT scan, with repeated drainage procedures if residual collection persists.

## 4 Discussion

This national survey, conducted in collaboration with three surgical societies, provides an overview of the current practice patterns in France regarding the diagnosis and management of AL following LCA or CAA with diverting ileostomy. Currently, there is no consensus or official guideline in France to assist practitioners in managing these cases, leading to significant variability in treatment approaches across centers. To our knowledge, this is the first study of this scale to systematically document current practices and to use these findings as the basis for proposing a standardized management algorithm not only for diagnosis but also for therapeutic management of colorectal or coloanal fistula. Although this consensus cannot be considered a formal recommendation, given that it does not necessarily reflect actual clinical practice, it serves as a foundation for developing a standardized consensus‐based algorithm, which could guide the design of a prospective randomized controlled trial to validate the proposed algorithm.

Despite ongoing advancements in AL prevention after rectal cancer surgery, including the use of indocyanine green angiography and the addition of oral antibiotics to mechanical bowel preparation, the incidence of AL remains as high as 15% even in experienced centers [6, pp. 6, 7]. The management of AL has remained relatively unchanged for several years, with salvage Hartmann's procedure continuing to be the most common option, even when redo coloanal or colorectal anastomosis is feasible [6, 7, 17, 31, 32, 33, 34, 35, 36]. However, this approach carries a 50% risk of resulting in a permanent stoma. Therefore, in the absence of generalized peritonitis, the optimal treatment should aim to rapidly control perianastomotic sepsis while preserving the possibility of intestinal continuity restoration [37, 38].

Early diagnosis is critical for anastomosis preservation [18, 39]. Clinical and biochemical monitoring during the early postoperative period plays a key role in guiding patient‐specific treatment. This study has established a hierarchy of clinical warning signs, including fever, tachycardia, and diffuse abdominal pain, that should prompt further diagnostic investigation. Fever and tachycardia were included in the original Dutch leakage (DULK) score, which predicts AL with a sensitivity of 97% and a negative predictive value of 99.5%. However, the modified DULK score replaced these parameters with respiratory rate, alongside clinical deterioration, pain (other than wound pain), and CRP levels [40, 41]. In our survey, oxygen saturation was favored over respiratory rate, as it was considered a more sensitive indicator of severe postoperative complications. Interestingly, unlike the modified DULK score, respiratory distress was not considered by the surveyed surgeons as a determining factor for AL diagnosis.

Conversely, more than 96% of participants reported routinely using serial CRP measurements rather than relying on hyperleukocytosis or thrombocytosis monitoring. The high specificity of CRP, demonstrated in numerous studies, along with its simplicity of use and low cost, makes it a biomarker widely appreciated by the surgical community [42, 43, 44, 45]. It has been demonstrated by Singh et al. that a CRP level < 170 mg/L on POD 3 and < 150 mg/L on POD 5 had an excellent negative predictive value of approximately 97% but a poor positive predictive value of only around 20% [46]. In contrast, it has been reported that patients with symptomatic anastomotic leaks had mean CRP values ranging from 230 to 255 mg/L between POD 1 and POD 5 [47]. Based on these findings, we proposed a CRP cutoff of 250 mg/L between POD 1 and 4 as a clinically relevant indicator to trigger further diagnostic investigations. This important point has been discussed in the revised version of manuscript. Only 20% of respondents reported measuring procalcitonin (PCT) in suspected AL cases, despite its well‐documented diagnostic value in the literature [45, 48, 49, 50, 51, 52].

This study is the first to integrate radiological findings into structured decision‐making algorithm. Participants considered contrast enhanced CT scans to be reliable only from postoperative day 3 onward, likely to minimize false positives even though contrast enhancement defect may be detected earlier [47]. Radiological signs, such as perianastomotic fluid collection, extraluminal air bubbles, or focal enhancement defect in the colonic wall, reached consensus as strong indicators of AL [53]. Interestingly, rectal contrast did not achieve consensus as indirect radiological findings were deemed sufficient for AL diagnosis. Nevertheless, the presence of contrast extravasation confirmed the AL for 88% of respondents. Few studies have directly compared CT scans with and without rectal contrast administration, but existing evidence suggests that contrast enema adds diagnostic value primarily when the initial contrast‐enhanced CT scan is negative despite strong clinical suspicion of AL [54, 55].

In cases of perianastomic fluid collection, most surgeons considered AEG to be essential for both diagnostic confirmation and therapeutic intervention, often obviating the need for rectal contrast administration. Therefore, AEG is pivotal in both diagnosis and treatment, allowing direct anastomosis assessment and simultaneous transanal drainage. In some cases, fluid collection optimal drainage requires widening of the fistula opening. This maneuver may be likely preferable to leaving an undrained abscess. Unlike image‐guided percutaneous drainage, transanal drainage allows for direct debridement and fistula widening, which may explain why it was favored by the majority of respondents in this survey. Although effective in clearing perianastomotic sepsis, widening of the fistula opening may increase the risk of anastomotic stricture. To mitigate this risk, negative pressure endoluminal therapy has been proposed as a promising adjunctive treatment [19, 27, 56, 57, 58, 59]. Negative pressure endoluminal therapy promotes wound contraction and drainage while preserving anastomotic integrity, making it a technique that is gaining increasing clinical adoption. Most of participants considered drainage as optimal when follow‐up imaging confirms the absence of a residual perianastomotic fluid collection.

One limitation of this survey is the lack of consideration for emerging endoscopic therapies, such as fibrin glue application, stenting, or clip placement [20, 57, 60]. Additionally, the role of the minimally invasive approach for transabdominal reoperation was not addressed, despite encouraging data supporting its use [61, 62]. Another potential limitation is the representativeness of the survey sample. Although estimates suggest that over 1000 general surgeons practice in France as of in 2010 [63, 64], this study reflects the perspectives of a subset of experienced surgeons affiliated with surgical associations, which may not fully represent nationwide practice patterns. Furthermore, this survey exclusively captured the views of surgeons, without incorporating the perspectives of radiologists and endoscopists, whose input could further refine the proposed algorithm. Finally, although the survey focused specifically on AL management, it did not address other perianastomotic complications, such as fistulas, occurring at the tip of the J‐pouch or in side‐to‐end anastomoses, conditions that, although less common, warrant further investigation.

## 5 Conclusion

The survey emphasized the importance of performing a CT scan from postoperative day 3 onward, particularly in the presence of clinical or biochemical indicators suggestive of an AL, such as elevated CRP level, fever, tachycardia, or diffuse abdominal pain. Notably, rectal contrast administration was deemed unnecessary for the first‐line diagnostic assessment. This study also highlighted the critical role of early aggressive management, with an anal examination under general anesthesia serving as both a key diagnostic and therapeutic procedure. Ultimately, this survey established a consensus‐based standardized management algorithm for coloanal or colorectal anastomotic leaks, which could be further evaluated in a controlled trial in the near future to assess its impact on mitigating the short‐term and long‐term consequences of anastomotic leaks.

## Author Contributions

**Clément Pastier:** investigation, writing – original draft, visualization, formal analysis. **Wafa Ben Hmida:** investigation. **Jérémie H. Lefèvre:** methodology, supervision. **Quentin Denost:** methodology, supervision. **Lilian Schwarz:** methodology, supervision. **Stéphane Berdah:** methodology, supervision. **Eddy Cotte:** methodology, supervision. **Mehdi Karoui:** methodology, supervision. **Léon Maggiori:** methodology, supervision. **Solafah Abdalla:** conceptualization, investigation, funding acquisition, writing – original draft, methodology, validation, visualization, writing – review and editing, software, formal analysis, project administration, data curation, supervision, resources. **Antoine Brouquet:** conceptualization, investigation, funding acquisition, writing – original draft, methodology, validation, visualization, writing – review and editing, project administration, formal analysis, software, data curation, supervision, resources. **Stéphane Benoist:** conceptualization, investigation, funding acquisition, writing – original draft, methodology, validation, visualization, writing – review and editing, project administration, formal analysis, software, data curation, supervision, resources.

## Ethics Statement

Health Research Authority approval was sought for this study but found not to be required.

## Conflicts of Interest

The authors declare no conflicts of interest.

## Supporting information

Figure S1

## Data Availability

The data that support the findings of this study are available from the corresponding author upon reasonable request.
