# Supplementary material for: Management of Low Colorectal/Coloanal Anastomotic Leak: Results of a French National Intergroups Practice Survey (FRENCH‐GRECCAR‐SFCD)
Source: World J Surg. 2025 May 21;49(7):1747–56. doi: 10.1002/wjs.12634 (PMC12282568; doi:10.1002/wjs.12634)
Supplement: Supplementary file 1 — Figure S1 [file WJS-49-1747-s001.docx]

**1. Which scientific society are you affiliated with?**

A. FRENCH

B. GRECCAR

C. SFCD

**2. How many years have you been practicing as an attending surgeon?**

A. < 5 years

B. 5-10 years

C. > 10 years

**3. Do you routinely perform serial C-reactive protein measurements after rectal surgery with a diverted low anastomosis?**

A. Yes

B. No

**4. In the event of isolated fever of 38.5°C between postoperative days (POD) 1 and 3, what measures do you systematically implement?**

A. Continued monitoring with a follow-up assessment and infectious workup at 24 hours

B. Anal examination under general anesthesia

C. Abdominopelvic CT scan within 24 hours

D. Empirical broad-spectrum antibiotic therapy (piperacillin-tazobactam)

E. Bedside rectal examination (without general anesthesia)

F. Other, provide details:

*Remarks: …………………………………………………………….*

**5. If C-reactive protein exceeds 250 mg/L between POD 1 and 4, what measures do you systematically implement?**

A. Continued monitored surveillance with reassessment at 24 hours

B. Anal examination under general anesthesia

C. Abdominopelvic CT scan within 24h

D. Empirical broad-spectrum antibiotic therapy (piperacillin-tazobactam)

E. Bedside rectal examination (without general anesthesia)

**6. After POD5, if CRP > 150 mg (isolated), what measures do you systematically implement?**

A. Continued monitored surveillance with reassessment at 24 hours

B. Anal examination under general anesthesia

C. Emergency abdominopelvic CT scan

D. Empirical broad-spectrum antibiotic therapy (piperacillin-tazobactam)

E. Bedside rectal anastomosis examination (without general anesthesia)

**7. In case of purulent discharge via drain or anus, what measures do you systematically implement?**

A. Continued monitored surveillance with reassessment at 24 hours

B. Anal examination under general anesthesia

C. Emergency abdominopelvic CT scan

D. Empirical broad-spectrum antibiotic therapy (piperacillin-tazobactam)

E. Bedside rectal anastomosis examination (without general anesthesia)

**8. Rank the following symptoms in order of importance as suggestive signs of an anastomotic fistula.**

A. Left iliac fossa abdominal pain

B. Diffuse abdominal pain

C. Anal pain

D. Fever $\geq$ 38.5°C

E. Tachycardia > 100 bpm

F. Ileus

G. Confusional state

H. Oxygen desaturation < 94%

**9. Rank the following blood test signs in order of importance as suggestive signs of a fistula.**

A. Leukocytosis > 12x10^9^/mm^3^

B. CRP > 250 mg/mL

C. Thrombocytosis > 600x10^9^/L

**10. Before which postoperative day do you consider a CT scan unnecessary for the diagnosis of an anastomotic fistula?**

A. POD2

B. POD3

C. POD4

D. POD5

E. Other, provide details:

*Remarks: …………………………………………………………….*

**11. In case of abdominopelvic CT scan for suspected fistula, do you systematically perform rectal contrast administration?**

A. Yes

B. No

**12. What radiological signs lead you to diagnose an anastomotic fistula?**

A. Extraluminal air bubbles

B. Perianastomotic fluid collection

C. Uncollected perianastomotic effusion

D. Contrast extra-anastomotic leakage

E. Focal enhancement defect in the colon wall at or upstream of the anastomosis

**13. In case of extraluminal air bubbles without other radiological signs, what management do you consider?**

A. Antibiotic therapy alone

B. Anal examination under general anesthesia ± transanal drainage

C. Image-guided percutaneous drainage

D. Surgical exploration via abdominal approach

E. Continued surveillance

F. Other, provide details:

*Remarks: …………………………………………………………….*

**14. In case of a perianastomotic fluid collection, what management do you consider?**

A. Antibiotic therapy alone

B. Anal examination under general anesthesia ± transanal drainage

C. Image-guided percutaneous drainage

D. Surgical exploration via abdominal approach

E. Other, provide details:

*Remarks: …………………………………………………………….*

**15. In case of uncollected perianastomotic effusion without extraluminal air bubbles, what management do you consider?**

A. Antibiotic therapy alone

B. Anal examination under general anesthesia ± transanal drainage

C. Image-guided percutaneous drainage D. Surgical exploration via abdominal approach

E. Continued surveillance

F. Other, provide details:

*Remarks: …………………………………………………………….*

**16. In case of contrast extra-anastomotic leakage, what management do you consider?**

A. Antibiotic therapy alone

B. Anal examination under general anesthesia ± transanal drainage

C. Image-guided percutaneous drainage

D. Surgical exploration via abdominal approach

F. Other, provide details:

*Remarks: …………………………………………………………….*

**17. In case of a focal enhancement defect in the colon wall upstream of the anastomosis, what management do you consider?**

A. Antibiotic therapy alone

B. Anal examination under general anesthesia ± transanal drainage

C. Image-guided percutaneous drainage

D. Surgical exploration via abdominal approach

F. Other, provide details:

*Remarks: …………………………………………………………….*

**18. When both are feasible, which drainage approach do you prefer,**

A. Image-guided percutaneous drainage

B. Transanal drainage

C. Other, provide details:

*Remarks: …………………………………………………………….*

**19. In case of a confirmed fistula during anal orendoscopic examination under general anesthesia, what type of drainage do you use?**

A. Passive drainage (transanal Foley catheter)

B. Passive drainage (transanal Foley catheter) + irrigation drain

C. Collection evacuation with suture orclip

D. Endoluminal vacuum therapy (Endosponge)

E. Other, provide details:

*Remarks: …………………………………………………………….*

**20. In case of a treated fistula, what duration of antibiotic therapy do you usually prescribe?**

A. 5 days

B. 7 days

C. 10 days

D. 15 days

E. Other, provide details:

*Remarks: …………………………………………………………….*

**21. In case of transanal or radiological drainage, when do you remove the drainage system?**

A. After more than 2 days

B. After more than 3 days

C. After more than 5 days

D. When drainage output is < 10 cc per 24 hours

E. When the drain stops completely

F. When the drainage becomes clear

G. When a control imaging shows no residual collection

H. As soon as the cavity has decreased in size

**22. In case of Endosponge drainage, how many sponge changes do you perform at a minimum?**

A. 1 change

B. 2 changes

C. 3 changes

D. 4 changes

E. 5 changes

F. Other, provide details:

*Remarks: …………………………………………………………….*

**23. In case of Endosponge drainage, how many changes do you perform at a maximum?**

A. 1 change

B. 2 changes

C. 3 changes

D. 4 changes

E. 5 changes

F. Other, provide details:

*Remarks: …………………………………………………………….*

**24. From what fistula size do you consider transanal or image-guided percutaneous drainage to be ineffective?**

A. ¼ of the anastomotic circumference

B. ½ circumference

C. ¾ circumference

D. Complete dehiscence

E. Never

**25. What is your usual practice after removal of the transanal drain?**

A. No specific action

B. Replacement with a soft drain for a few days

C. Syringe irrigation

D. Simple dry dressing

**26. In case of suspected anastomotic fistula, do you measure procalcitonin (PCT)?**

A. Yes

B. No
